# Decline in oral function contributes to decreased activities of daily living at discharge in elderly patients with heart failure

**DOI:** 10.1371/journal.pone.0323806

**Published:** 2025-05-28

**Authors:** Misaki Nakamura, Kanako Yamamoto, Shinichi Nozaki, Takahiro Saeki, Wataru Omi, Chieko Kato, Masaru Inoue, Tomoya Harada, Satoru Sakagami

**Affiliations:** 1 Department of Dentistry and Oral Surgery, NHO Kanazawa Medical Center, Kanazawa, Ishikawa, Japan; 2 Department of Cardiology, NHO Kanazawa Medical Center, Kanazawa, Ishikawa, Japan; Niigata University of Health and Welfare: Niigata Iryo Fukushi Daigaku, JAPAN

## Abstract

Low body weight is associated with decreased capacity for performing activities of daily living (ADLs) after discharge in patients with heart failure (HF). In particular, malnutrition and low body mass index (BMI) are considered poor prognostic factors. In this study, we aimed to examine the relationship between oral function and low body weight as well as the ability to perform ADLs in older patients with HF. To this end, we retrospectively examined patients with HF aged 75 years or older who had undergone oral function assessments. We examined various factors including age, sex, BMI, brain natriuretic peptide (BNP) levels, left ventricular ejection fraction (LVEF), Oral Health Assessment Tool-Japanese version (OHAT-J) scores, Barthel Index (BI) scores, and length of hospital stay. Patients were categorized into two groups based on their OHAT-J scores (OHAT-J ≤ 2 or OHAT-J ≥ 3), and their characteristics, including individual survey items, were compared. Additional correlation analyses were performed for BI at discharge. We found a negative correlation between the total OHAT-J score and BI at discharge. Specifically, lower BIs at discharge were observed in the group with poor scores for the items “lips,” “saliva,” and “dentures.” Oral dysfunction was associated with decreased ability to perform ADLs at discharge in older patients with HF. Thus, interdisciplinary interventions targeting oral health may improve the prognosis of patients with HF.

## Introduction

The number of heart failure (HF) patients is estimated to be 64 million worldwide and is increasing due to aging populations and improved survival rates. In recent years, the growing number of HF patients in Japan has also become a major concern [1–3]. Japan, in particular, has one of the highest aging rates in the world, and the increase in HF among older patients is especially pronounced. Maintaining activities of daily living (ADL) is crucial for preventing HF exacerbation-related readmissions and improving quality of life [1]. However, patients with acute HF and low body weight have been reported to have a decreased ability to perform ADL at discharge [4]. In HF patients, poor nutrition and low body mass index (BMI) are considered adverse prognostic factors, and it has been reported that not only severe obesity but also low body weight is associated with higher mortality rates [5–7]. A comparison of HF patients aged 65 and older in the United States and Japan found that the length of hospital stay was longer in Japan, and a higher percentage of patients received cardiac rehabilitation within three months of hospitalization [8]. Even with longer hospital stays, to prevent a decline in ADL at discharge, rehabilitation is conducted early during hospital stay by physical therapists using the acute phase mobilization program for acute HF patients [9]. In hospitalized older patients, a decline in oral function has been noted along with overall health deterioration, with correlations to sarcopenia, malnutrition, and increased mortality rate [10–12]. Furthermore, patients with severe periodontal disease have a higher cardiovascular risk, higher prevalence of HF, and increased mortality rate from cardiovascular diseases [13–16]. In Japan, where rapid aging is occurring compared to the rest of the world, the number of older HF patients is sharply increasing, and it has been pointed out that the decline in oral function is affecting malnutrition and HF [1,5,14,15]. Therefore, dental hygienists who have obtained certifications in HF care participate in the multidisciplinary HF team, conduct oral function assessments, and provide guidance for maintaining oral health. In this study, we aimed to investigate the effect of oral function decline in older patients hospitalized due to HF on body weight, ADL, length of hospital stay, and mortality rate.

## Materials and methods

### Ethical considerations

This study was approved by the Ethical Review Board of NHO Kanazawa Medical Center (R04-068). Since this was a retrospective study, the requirement for informed consent was waived. Information regarding the purpose and implementation of the research was disclosed on the hospital’s website, ensuring opportunities for refusal wherever necessary. This study was reported in accordance with the Strengthening the Reporting of Observational Studies in Epidemiology (STROBE) statement.

### Study population

This retrospective study included 69 patients with HF aged 75 years or older who were admitted to the cardiology ward of our hospital between April 1, 2022, and December 31, 2022, and were referred for oral function management to dental hygienists. Patients under 75 years old with HF or those who were not referred for oral function management by the attending physician were not included in the study.

### Data source

Data were extracted from electronic medical records between March 24, 2023, and June 30, 2023, for a retrospective analysis. The analyzed items included Oral Health Assessment Tool – Japanese version (OHAT-J) scores, cardiac rehabilitation, sex, age, left ventricular ejection fraction (LVEF), BMI at admission, brain natriuretic peptide (BNP) levels at admission, Barthel Index (BI) scores at admission, death, hospital stay days, BMI at discharge, BNP levels at discharge, BI scores at discharge, and each of eight OHAT-J assessment item scores.

#### Oral health assessment tool - Japanese version.

The OHAT-J [17] is a simplified oral screening score that can be used by anyone, including non-dental healthcare providers. It is based on the OHAT assessment sheet [18] reported by Chalmer, and was translated to Japanese. The OHAT-J assesses eight items, including the lips, tongue, gums and tissues, saliva, natural teeth, dentures, oral cleanliness, and dental pain, each of which is rated as healthy or unhealthy on a scale of 0–2 points. The total score ranges from 0 to 16 points, with higher scores indicating greater oral dysfunction.

In our hospital, the OHAT-J is used for oral function assessments and HF care guidance in older patients. Oral function assessments were conducted by a dental hygienist in the early phase of hospitalization (within one week of admission, except in eight cases).

#### Barthel index.

The BI is an ADL assessment tool [19] which comprises 10 items: eating, transferring from a wheelchair to a bed, grooming, toileting, bathing, walking, climbing stairs, dressing, bowel control, and urinary control. Each item is scored on a scale of 0, 5, 10, or 15 points, with maximum scores ranging from 5 to 15 points depending on the item. Total scores range from 0 to 100 points, with higher scores indicating higher levels of independence. The BI was extracted from diagnosis procedure combination (DPC) data or rehabilitation implementation plans. DPC is a coding system used in Japan’s medical reimbursement system, indicating combinations of medical procedures used. It is inputted with necessary items depending on the illness.

### Sample size calculation

Owing to the lack of previous research on the decline in oral function and ADL in older patients with HF, the mean difference and standard deviation were determined by referring to the BI values in older patients compared by nutritional disorders or post-stroke swallowing function [20–22]. For the comparison of the mean values between the two groups, the sample size was calculated using a mean difference of 15 between the two groups, a common standard deviation of 21, an alpha error of 0.05, a power of 0.80, and a sample size ratio of 1:1 between the two groups. Using statistical software (EZR software version 1.52, Chugai Igakusha, Tokyo, Japan) and a systematic sampling method, the sample size was calculated to be 31 per group [23]. The hospitalization rate due to HF at our institution is approximately 80% for those aged 75 and older, with a mortality rate of about 5% for the same age group (not publicly available). In all patients, including those under 75 years old, the calculated target sample size is approximately 82, determined by the formula: (31 × 2)÷0.95 ÷ 0.8. Oral function management for HF patients is requested at a rate of approximately 10 patients per month (not publicly available), requiring an observation period of about 9 months.

The power was calculated based on the results obtained from a post-hoc test. For the two groups with sample sizes of 33 and 36, a mean difference of 15.8, a standard deviation of 28.85, and an alpha error of 0.05, the power was 0.623 when a two-sided test was performed.

#### Relationship between the total OHAT-J and patient background information and indication for admission.

The patients were divided into two groups depending on their total OHAT-J score (OHAT J ≤ 2 or OHAT-J ≥ 3) [11]. Cardiac rehabilitation, sex, age, LVEF, BMI at admission, BNP levels at admission, and BI at admission were compared between the two groups.

#### Comparison and correlation with total OHAT-J.

The patients were divided into two groups depending on their total OHAT-J score (OHAT J ≤ 2 or OHAT-J ≥ 3) [11]. Death, hospital stay days, BMI at discharge, BNP levels at discharge, and BI at discharge were compared between the two groups. The correlation between the total OHAT-J score and each survey (hospital stay days, BMI at discharge, BNP levels at discharge, and BI at discharge) item was assessed.

#### Relation between the eight OHAT assessment items and discharge BI.

Additional analyses were conducted for the discharge BI, which demonstrated a correlation with the total OHAT-J score. Based on the correlation between the total OHAT-J score and the BI at discharge, the relationship between the eight individual OHAT-J assessment items and the BI at discharge was additionally evaluated. Scores for the eight individual OHAT-J assessment items were categorized as healthy (score of 0) or unhealthy (score of 1 or 2) and compared with the mean discharge BI.

### Statistical analysis

All statistical analyses were performed using EZR software version 1.52 (Chugai Igakusha, Tokyo, Japan) [23]. EZR is a statistical software that extends the functionalities of R and its commander. Missing values were observed for LVEF in one case, discharge BNP in four cases, admission BMI in one case, discharge BMI in one case, and discharge BI in two cases (due to patient death). An available case analysis was conducted. Statistical significance was considered at p < 0.05.

#### Relationship between total OHAT-J score and each survey item.

The patients were divided into two groups depending on their total OHAT-J score (OHAT-J ≤ 2 or OHAT-J ≥ 3). Patient characteristics (including individual survey items) were compared between the two groups. Fisher’s exact tests were used to compare proportions, and t-tests were used to compare means. The correlation between the total OHAT-J score and each survey item was assessed using Pearson’s correlation coefficient tests. Additionally, a univariate regression analysis was conducted to assess the correlation between the BI at discharge and total OHAT-J score.

#### Relationship between the eight OHAT-J assessment items and BI at discharge.

Additional analyses were conducted for the BI at discharge, which demonstrated a correlation with the total OHAT-J score. Scores for the eight individual OHAT-J assessment items were categorized as healthy (score of 0) or unhealthy (score of 1 or 2) and compared with the mean discharge BI. One-sample Kolmogorov-Smirnov tests were conducted to assess for normality. If the number of cases per group for each item was N < 30 with a non-normal distribution, Welch’s t-tests were used; if N ≥ 30 or the distribution was normal, t-tests were applied. The “lips” item was compared between groups using Welch’s tests, while the other seven items were compared using t-tests. Multiple regression analysis (stepwise variable elimination using p-values) was then performed to identify the associations between the eight OHAT-J assessment items and discharge BI.

## Results

### Relationship between the total OHAT-J score and patient background information and indication for admission

Of 69 participants, 36 had a total OHAT-J ≤ 2 and 33 a total OHAT-J ≥ 3. No significant differences were observed between the groups in terms of patient characteristics or BI at admission. Additionally, LVEF (%) was 51.1 ± 14.9 in the OHAT-J ≤ 2 group and 56.0 ± 13.4 in the OHAT-J ≥ 3 group (P = 0.162), and BNP at admission (pg/mL) was 779.5 ± 655.9 in the OHAT-J ≤ 2 group and 646.0 ± 544.8 in the OHAT-J ≥ 3 group (P = 0.364), with no significant differences in cardiac function or HF markers. Cardiac rehabilitation was used in 34 patients (94.4%) in the OHAT-J ≤ 2 group and 32 (97.0%) in the OHAT-J ≥ 3 group (Table 1).

**Table 1:** Comparisons of patient characteristics based on OHAT-J.

Variables	OHAT-J 0–2 (n = 36)	OHAT-J ≥ 3 (n = 33)	*p*-value
males, n (%)/females, n (%)	14 (38.9)/22 (61.1)	16 (48.5)/17 (51.5)	0.472
age, years, mean±SD	87.0 ± 5.5	88.1 ± 6.8	0.464
BMI at admission, kg/m^2^, mean±SD	22.3 ± 3.2	21.5 ± 3.1	0.344
BI at admission, point, mean±SD	59.3 ± 40.8	53.2 ± 40.5	0.534

OHAT-J total score 0–2 group vs OHAT-J total score ≥3 group using t-test. OHAT-J, oral health assessment tool-Japanese version; SD, standard deviation; BMI, body mass index; BI, Barthel index.

#### Comparison of groups based on the total OHAT-J score (OHAT-J ≤ 2, OHAT-J ≥ 3).

There were no significant differences between the groups regarding the length of stay, discharge BMI. BNP at discharge was 309.3 ± 244.9 pg/mL in the OHAT-J 0–2 group and 324.5 ± 517.0 pg/mL in the OHAT-J ≥ 3 group (p = 0.878), indicating no difference in the degree of HF between the two groups. However, the discharge BI was significantly lower in the OHAT-J ≥ 3 group (average score (± standard deviation) of 68.6 ± 34.2) when compared with the OHAT -J ≤ 2 group (84.4 ± 23.5) (*p* = 0.029). Two deaths (6.1%) were recorded in the OHAT-J ≥ 3 group (Table 2).

**Table 2:** Comparisons of outcomes based on OHAT-J.

Variables	OHAT-J 0–2 (n = 36)	OHAT-J ≥ 3 (n = 33)	*p-*value
Death, n (%)	0 (0.0)	2 (6.1)	0.225
Hospital stay days, days, mean±SD	24.9 ± 20.3	18.9 ± 8.2	0.120
BMI at discharge, kg/m^2^, mean±SD	20.2 ± 4.0	19.6 ± 3.1	0.465
BI at discharge, point, mean±SD	84.4 ± 23.5	68.6 ± 34.2	0.029^*^

* *p* < 0.05, OHAT-J total score 0–2 group vs OHAT-J total score ≥3 group using t-test. OHAT-J, oral health assessment tool-Japanese version; SD, standard deviation; BMI, body mass index; BI, Barthel index.

#### Correlation with the total OHAT-J score.

**Fig 1:**
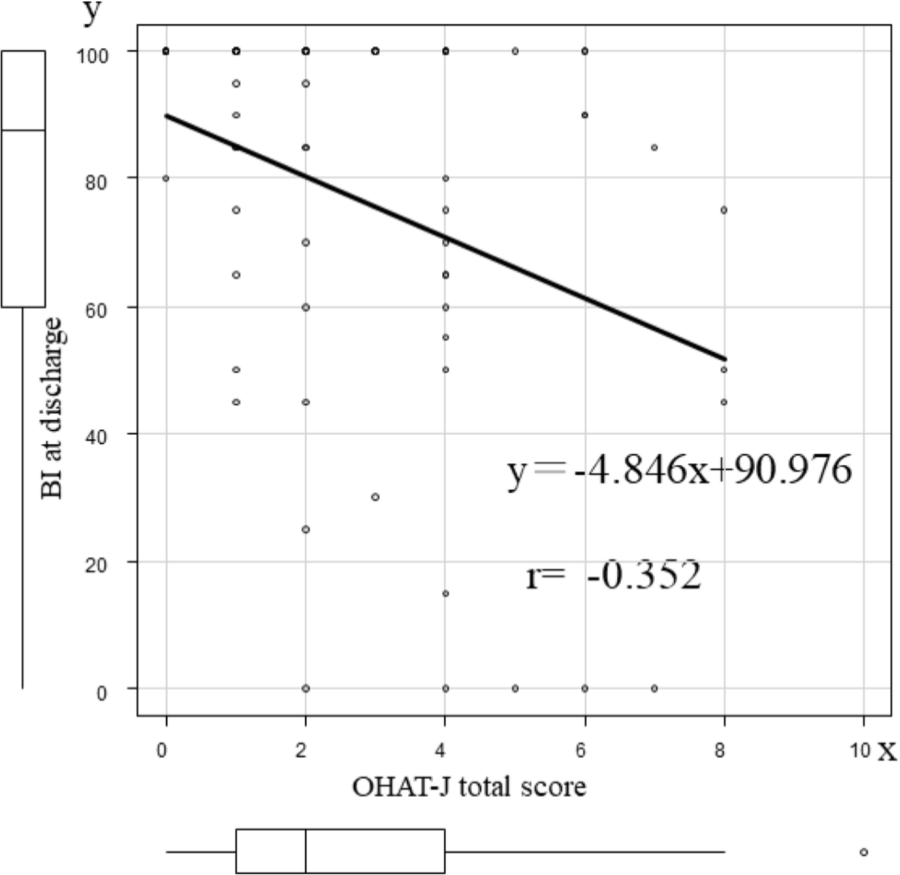
Correlation between the total OHAT-J score and BI at discharge. Correlation diagram and univariate analysis using Pearson’s product-moment correlation showed a weak negative correlation between the total OHAT-J score and discharge BI, with a correlation coefficient of -0.352. OHAT-J, oral health assessment tool-Japanese version; BI, Barthel index.

No correlation was observed between the total OHAT-J score and length of stay (r = -0.134, *p* = 0.273), discharge BMI (r = -0.125, *p* = 0.310), or discharge BNP (r = 0.042, *p* = 0.739). The correlation coefficient between the total OHAT-J score and discharge BI was -0.352, indicating a weak negative correlation (*p* = 0.004). Based on the simple regression analysis between the total OHAT-J score and discharge BI, the following regression equation was obtained: discharge BI = 90.976 - 4.846 × “OHAT-J total score” (Table 3, Fig 1).

**Table 3:** Pearson’s product-moment correlation between outcomes and the OHAT-J total score.

Variables	r	*p*-value
Hospital stay (days)	–0.134	0.273
BMI at discharge	–0.125	0.310
BI at discharge	–0.352	0.004^*^

* *p* < 0.05, using Pearson’s product-moment correlation. OHAT-J, oral health assessment tool-Japanese version; BMI, body mass index; BI, Barthel index.

#### Relationship between the eight OHAT-J assessment items and discharge BI.

**Fig 2:**
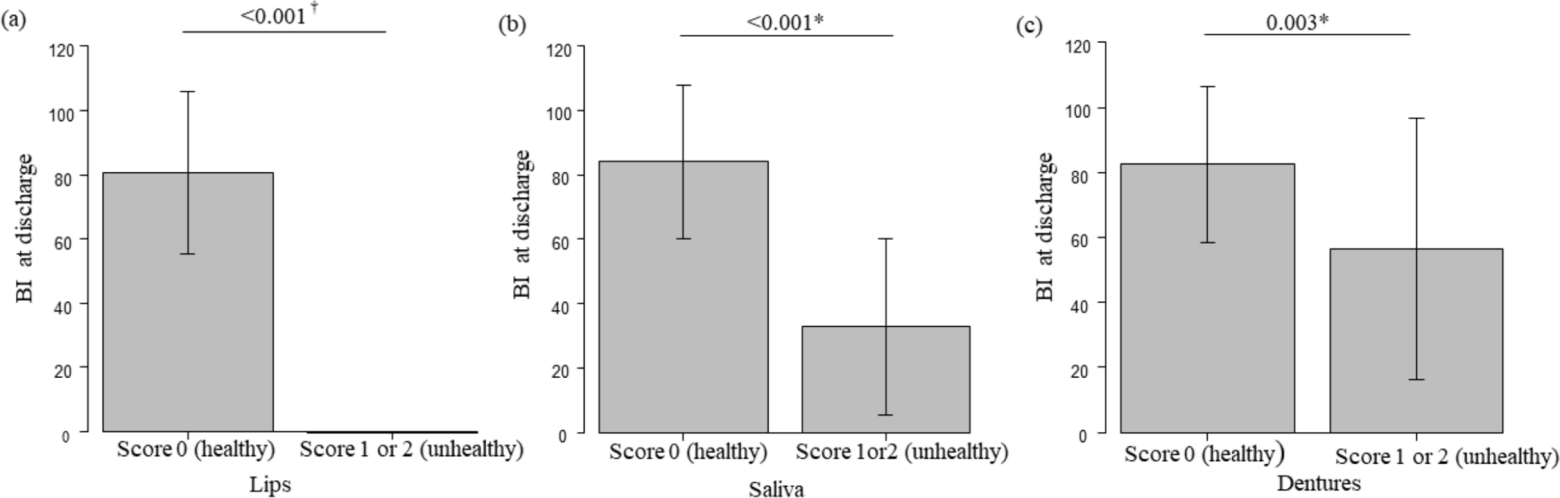
(a)(b)(c). Relationship between the OHAT-J score for “lips” or “saliva” or “dentures” and the BI at discharge. The BI at discharge were significantly lower in the unhealthy (1–2 points) group for”(a) lips,” “(b)saliva,” and “(c)dentures” when compared to those in the healthy (0 points) group. ^†^*p* < 0.05, OHAT-J score 0 (healthy) group vs OHAT-J score 1 or 2 (unhealthy) group using Welch test. * *p* < 0.05, OHAT-J score 0 (healthy) group vs OHAT-J score 1 or 2 (unhealthy) group using t-tests. OHAT-J, oral health assessment tool-Japanese version; BI, Barthel index.

Among the eight OHAT-J assessment items, the discharge BI scores were significantly lower in the unhealthy (1–2 points) group for “lips,” “saliva,” and “dentures” when compared to those in the healthy (0 points) group. Multiple regression analysis revealed that these three items (“lips,” “saliva,” and “dentures”) were related to the discharge BI, and the following multiple regression equation was obtained: discharge BI = 88.112–10.085 × “OHAT-J dentures score” −30.273 × “OHAT-J saliva score” −61.206 × “OHAT-J lips score” (Table 4, Fig 2a–c).

**Table 4:** BI at discharge based on oral health by category of the OHAT-J.

Category	OHAT-J 0 (healthy)	OHAT-J 1 or 2 (unhealthy)	*p*-value
	BI at discharge, mean (±SD)	BI at discharge, mean (±SD)	
Lips	80.7 ± 25.2	00.0 ± 00.0	<0.001^†^
Tongue	79.1 ± 28.9	56.7 ± 34.6	0.079
Gums and oral tissue	82.0 ± 27.4	68.3 ± 32.6	0.072
Saliva	84.0 ± 23.8	32.8 ± 27.4	<0.001*
Natural teeth	75.6 ± 32.5	79.6 ± 25.1	0.599
Dentures	82.5 ± 24.1	56.4 ± 40.2	0.003*
Cleanliness	77.1 ± 33.3	77.1 ± 28.9	0.996
Dental pain	77.7 ± 29.2	67.5 ± 42.5	0.515

† *p* < 0.05, OHAT-J score 0 (healthy) group vs OHAT-J score 1 or 2 (unhealthy) group using Welch test. * *p* < 0.05, OHAT-J score 0 (healthy) group vs OHAT-J score 1 or 2 (unhealthy) group using t-test. OHAT-J, oral health assessment tool-Japanese version; BI, Barthel index.

## Discussion

HF patients are increasing globally with aging, and in Japan, where aging has progressed rapidly, the prevalence of HF among older patients has markedly increased, leading to what is referred to as the “heart failure pandemic” [1]. Patients with HF often experience a decline in their ability to perform ADLs owing to recurrent hospitalizations, which is of significant concern. Ogawa et al. reported that among patients hospitalized for acute HF in Japan, those with a lower body weight experienced greater declines in their ability to perform ADLs during hospitalization, prolonging their length of stay [4]. Matsuo et al. showed that malnutrition in hospitalized older patients was associated with decreases in oral function, appetite, quality of life (QOL), and the ability to perform ADLs [10]. Therefore, in this study, we investigated whether worse oral function in older patients with HF is related to low body weight, decreased ability to perform ADLs, and prolonged hospitalization. As an evaluation item for ADL, besides the BI, the Functional Independence Measure (FIM) is well known, which includes cognitive function evaluation items in addition to motor function [24]. In this study, the BI was used as an ADL evaluation tool because it includes motor evaluation items equivalent to those of the FIM and is evaluated at the time of admission and discharge as a DPC evaluation item.

In older inpatients with various diseases, it has been reported that patients with an OHAT score of 3 or more have lower admission BI, longer hospital stays, and higher mortality rates compared to those with OHAT scores of 0 and 1–2 [11]. Therefore, in this study, we also divided the overall OHAT-J score into two groups for comparison: a group with an OHAT-J score of 0–2 and a group with an OHAT-J score of 3 or higher. There were no differences between the two groups in terms of sex, age, left ventricular function, BMI, BNP, or BI at admission. Additionally, there were no differences between the groups regarding the receipt of cardiac rehabilitation, with more than 90% of patients in both groups receiving it. This indicates that there was no significant relationship between oral function decline as measured by the OHAT-J score (classified into the two groups of 0–2 and 3 or higher) and patient background at admission, and that equivalent cardiac rehabilitation was provided in both groups to maintain ADL at admission. Mortality during hospitalization was observed only in the group with an OHAT-J score of 3 or higher, but there was no significant difference. In addition, there were no significant differences between the two groups in terms of the length of hospital stay, BMI at discharge, or BNP levels at discharge. Our results showing that prolonged hospital stays and increased mortality were not observed in the high OHAT-J group differ from those in the report by Maeda et al. [11]. In this study, the lack of difference in BI at admission might have affected the length of hospital stay and mortality. Additionally, older inpatients might have extended hospital stays owing to social backgrounds and discharge destinations, such as transfer to nursing care facilities or other convalescent hospitals if they cannot return home based on family support, ADL, or cognitive status, even after their HF conditions improve. This study did not investigate whether hospital stays were extended due to social backgrounds or discharge destinations, making it insufficient to evaluate the relationship between oral function and hospital stay duration.

At discharge, the BI improved compared to admission in both groups, regardless of oral function impairment. However, the improvement in BI was modest in the OHAT-J ≥ 3 group, and the discharge BI was significantly lower compared to the OHAT-J 0–2 group. Patients admitted due to acute HF exacerbation may have had difficulties performing ADLs such as toileting and walking due to shortness of breath or respiratory distress, leading to a decline in ADL capacity immediately after admission, which could have resulted in a lower BI at admission compared to their pre-admission ability. Although the decline in ADL ability due to acute HF exacerbation can improve with HF treatment [25] and cardiac rehabilitation [26,27], it may also worsen due to the progression of physical frailty. Since this study did not assess HF treatment or frailty, it is difficult to exclude the impact of physical frailty on the discharge BI. However, oral function impairment is associated with malnutrition [10,11], and multidimensional frailty, which evaluates physical, mental, nutritional, and socio-economic factors in elderly heart failure patients, is more predictive of mortality, loss of one or more basic ADLs, and hospital admissions compared to physical frailty alone [28]. Thus, oral function impairment may be involved in the decline of multidimensional frailty and may be related to a decrease in the discharge BI. Furthermore, although there was no correlation between the OHAT-J score and discharge BMI or discharge BNP, a weak negative correlation was observed between the OHAT-J score and discharge BI (r = -0.352, p = 0.004). This shows similar results to previous reports showing a negative correlation between the OHAT total score and FIM score in patients admitted to the rehabilitation department of a convalescent hospital [29]. The results of this study suggest that the early decline in oral function, independent of BMI decline and HF severity, is related to a decline in ADL at discharge. In the report by Maeda et al., although there was no difference in BMI in the group with a high OHAT-J score, malnutrition and low BI were observed [11], suggesting that differences in nutritional status may have influenced the results. However, since nutritional status was not evaluated in this study, it remains a subject for future research.

In the subgroup with impairments in “lips,” “saliva,” and “dentures” among the eight OHAT-J assessment items, there was a significant decrease in the BI at discharge, and multiple regression analysis indicated an association between certain items (“lips” and “saliva”) with lower BI at discharge. A score of 1–2 for “lips” and “saliva” in the OHAT-J indicates a dry mouth [17]. In patients with HF, the use of multiple medications, such as diuretics, calcium channel blockers, ACE inhibitors, and SGLT2 inhibitors, can induce dry mouth (drug-induced xerostomia) [30,31]. Furthermore, in older patients, polypharmacy for various conditions may be associated with declining oral function [32]. Additionally, patients with HF often have diabetes mellitus and are prone to dehydration [33]. Furthermore, patients with HF may experience salivary gland dysfunction [34]. As mentioned above, older patients with HF are prone to dry mouth, leading to higher OHAT-J scores for the “lips” and “saliva” items. Oral function impairment in hospitalized older patients has been associated with sarcopenia and malnutrition [12]. Oral dryness can lead to taste abnormalities and reduced appetite [35,36]. Although dietary intake and nutritional status were not assessed during admission, it is speculated that decreased oral intake may have led to the progression of malnutrition, resulting in a lower BI at discharge. Furthermore, an OHAT-J score of 1–2 points for “dentures” indicates poor or inadequate denture fitting [17]. In older individuals, a decrease in the number of teeth is associated with reduced bite force, diminished physical strength, and decreased mobility [37,38]. Edentulous older individuals who do not use dentures have been reported to have lower albumin levels and a decreased ability to perform ADLs when compared to denture users [39]. In the present study, high OHAT-J scores for dentures were associated with the BI at discharge, suggesting that reduced bite force and nutritional deficiencies may be related to the decline in ADLs [37–40].

From our findings, it is evident that oral function influences the decrease in the ability to perform ADLs associated with recurrent hospitalizations in patients with HF, suggesting the potential of oral health interventions for this issue. At our institution, we conducted an initial assessment of the oral cavity at admission and provided oral health management, particularly for addressing oral dryness and poor denture conditions, for older patients with HF.

Although it is impossible to compare cases where oral health management interventions were not performed, Suzuki et al. reported that interdisciplinary oral health management via a nutrition support team (NST) improved patients’ OHAT scores [41]. Furthermore, Huh et al. reported that oral care reduced the risk of HF in patients with type 2 diabetes [42]. Therefore, interdisciplinary interventions targeting oral health may prevent the onset of HF and improve its prognosis.

One of the limitations of this study is that there was no control group of older patients without HF. Therefore, the extent to which HF influenced the outcomes of this study is unclear in the context of the general population. Future studies should conduct a comparative analysis of older patients with HF and without HF. Furthermore, since the relationship between oral function impairment, dietary intake, nutritional assessment, and BI was not evaluated, additional investigations are needed. Additional studies are also needed to determine whether OHAT-J scores in the early stages of admission can predict the BI at discharge.

## Conclusions

In this study, a negative correlation was observed between the total OHAT-J score in the early stage of hospitalization and the BI at discharge in older patients with HF, indicating that declines in oral function were related to ADL performance at discharge.
